# Audit of Smoking Status Documentation and Smoking Cessation Interventions in Psychiatric Inpatients Department of Psychiatry, Gulab Devi Teaching Hospital, Lahore

**DOI:** 10.1192/bjo.2026.11841

**Published:** 2026-06-30

**Authors:** Muhammad Mansoor Ali, Syeda Areeba Siraj, Mudassar Ijaz, Ali Abbas Zahid, Maryam Arshad

**Affiliations:** 1 Al Aleem Medical Collge / Gulab Devi Teaching Hospital, Lahore, Pakistan; 2 Avicenna Hospital, Lahore, Pakistan

## Abstract

**Aims:**

To assess documentation of smoking status on admission, delivery of smoking cessation interventions to current smokers, and identification of gaps in referral and follow-up among psychiatric inpatients, followed by implementation of targeted interventions and evaluation of improvement. Et al Authors: 6, Dr Muhammad Zubair (email:), General Practitioner, Lahore, Pakistan

**Methods:**

A closed-loop prospective clinical audit was conducted in the Psychiatry Department >over two cycles in 2 months. A total of 60 psychiatric inpatients were included, with 30 patients in each phase. Pre-intervention data were collected from admission clerking notes and inpatient records to assess smoking status documentation, brief cessation advice, nicotine replacement therapy (NRT), referral to smoking cessation services, and follow-up planning. Interventions included staff education, reminders during admission clerking, and emphasis on documenting smoking cessation measures. Post-intervention data were collected using the same standards to assess improvement.

**Results:**

Pre-intervention (n=30): Smoking status was documented in 66.7% of patients. Among identified current smokers (n=12), brief cessation advice was provided in 33.3%, NRT was offered in 25%, referral to smoking cessation services occurred in 8.3%, and a documented follow-up plan was present in 16.7%. Post-intervention (n=30): Smoking status documentation improved to 93.3% (absolute improvement +26.6%). Among current smokers (n=13), brief cessation advice increased to 84.6% (+51.3%), NRT provision to 76.9% (+51.9%), referral to smoking cessation services to 53.8% (+45.5%), and documentation of follow-up plans to 69.2% (+52.5%).

**Conclusion:**

This closed-loop audit demonstrated that baseline documentation of smoking status and delivery of smoking cessation interventions in psychiatric inpatients were initially suboptimal. Following targeted educational and system-based interventions, improvement was observed across all assessed domains, including smoking status documentation (+26.6%), provision of brief cessation advice (+51.3%), offering or prescription of NRT (+51.9%), referral to smoking cessation services (+45.5%), and documentation of follow-up plans (+52.5%). These findings highlight that structured interventions can significantly improve preventive cardiovascular risk management in psychiatric inpatient settings. Regular re-auditing is recommended to sustain compliance and embed smoking cessation as a routine component of inpatient psychiatric care.

